# Cohort profile: the Maharashtra Anaemia Study 3 (MAS 3)—a maternal-child cohort study up to age 18 years in India

**DOI:** 10.1136/bmjopen-2025-104184

**Published:** 2025-10-28

**Authors:** Melissa T Benavente, Nophar Geifman, Sarah C Bath, Kourosh R Ahmadi, Andrew W Fogarty, Charles Marshall, Sumantra Ray, Laila J Tata, Chittaranjan Yajnik, Anand Ahankari

**Affiliations:** 1 Faculty of Health and Medical Sciences, University of Surrey, Guildford, UK; 2 Lifespan and Population Health Unit, School of Medicine, University of Nottingham, Nottingham, UK; 3 Wolfson Institute of Population Health, Queen Mary University of London, London, UK; 4 NNEdPro Global Institute for Food, Nutrition and Health, St John’s Innovation Centre, Cambridge, UK; 5 School of Biomedical Sciences, Ulster University, Coleraine, UK; 6 Centre for Perinatal Research, University of Nottingham, Nottingham, UK; 7 Diabetes Unit, King Edward Memorial Hospital Research Centre, Pune, India; 8 Faculty of Health, Psychology and Social Care, Manchester Metropolitan University, Manchester, UK

**Keywords:** Anaemia, Child, EPIDEMIOLOGY, Pregnancy, India

## Abstract

**Abstract:**

**Purpose:**

The Maharashtra Anaemia Study 3 (MAS 3) aims to (1) Investigate the nutritional, environmental, and economic impacts on haemoglobin concentration/anaemia, (2) Identify the underlying micronutrient causes of anaemia and (3) Investigate the association between anaemia and physical and cognitive development of Indian children during their first 18 years of life. This paper introduces the MAS 3 cohort, which consists of data collected from the participants in the prospective Pune Maternal Nutrition Study from the antenatal period to children at 18 years of age (1996–2014) in the Maharashtra state, India.

**Participants:**

Recruitment of 2466 married non-pregnant women, and their husbands, took place between June 1994 and April 1996 in six villages, approximately 50 km from Pune city in India. Women were followed up monthly to identify those who became pregnant. A total of 797 pregnant women were followed up for data collection at or near gestational week 18 and 28, with further data collection for women and children occurring within 72 hours of delivery, for both live and stillbirths. Of the 797 women, 710 were included in the MAS 3 cohort, and long-term follow-up of children occurred at 6 years, 12 years and 18 years of age.

**Findings to date:**

In the MAS 3 cohort, most mothers (73%) were aged between 18 and 25 years at the time of their final prepregnancy visit (baseline), and half (55%) belonged to families of middle-upper socioeconomic status (SES). At the children’s baseline (birth) visit, children had a mean birth weight of 2630 g (SD: 376), with one third (31%) of low birth weight. At the 6-year, 12-year and 18-year follow-up visits, data were available for 706 (99%), 689 (97%) and 694 (98%) children.

**Future plans:**

MAS 3 will be used to address a number of research objectives, including (1) Trends of haemoglobin and anaemia-related micronutrients from age 6 to 18 years, (2) Micronutrient causes of anaemia during childhood, (3) Prevalence and risk factors for maternal anaemia and childhood anaemia, (4) Impact of maternal anaemia on immediate birth outcomes and (5) Intergenerational risk factors associated with anaemia.

**STRENGTHS AND LIMITATIONS OF THIS STUDY**

- The study collected data on children within 72 hours of birth and every 6 years, until children were 18 years of age.
- Extensive data collection includes detailed anthropometry, medical history, dietary intake, cognitive development, hygiene practices and laboratory investigations.
- Gold-standard laboratory methods were used to measure haemoglobin and other anaemia-related micronutrients in a rural population.
- Recruitment occurred in six villages, approximately 50 km from Pune city in India, in a predominantly rural setting, which is unlikely to be representative of Maharashtra state or India as a whole.
- Due to the lack of formal education within the study population and high rates of illiteracy, all questionnaires were completed by field workers, potentially increasing the risk of interviewer bias, which should be considered.

## Introduction

 Anaemia is defined by a low concentration of haemoglobin in the circulating blood.^1^ For children, anaemia during childhood and adolescence is a serious concern since it can impair physical growth, affect cognitive development and result in increased morbidity from infectious diseases.^1 2^ During pregnancy, anaemia in the mother has been linked to an increased maternal morbidity and mortality, as well as adverse birth outcomes such as low birth weight (LBW).^3^ Risk factors for childhood anaemia include low socioeconomic status (SES), often indicated by household wealth or mother’s educational attainment or occupation, vegetarian diet, onset of menarche (girls) and maternal anaemia.^2 4 5^ The fifth national family health survey (NFHS-5) of India has reported high anaemia prevalence in children aged 6–59 months (67%), pregnant women aged 15–49 years (52%) and all women aged 15–49 years (57%).^2^ The NFHS, along with other nationally representative surveys conducted in India, such as the comprehensive national nutrition survey^6^ and district level household survey,^7^ is widely used to provide regular anaemia prevalence estimates at the national and state level. In doing so, these surveys provide policy makers with evidence as to the effectiveness of state and national anaemia prevention strategies. The high anaemia prevalence reported in NFHS-5 is despite a number of anaemia prevention strategies that have been implemented by the Indian government, starting as early as 1970 with the national nutritional anaemia prophylaxis programme^8^ and including more recent strategies such as Anaemia Mukt Bharat, which was implemented in 2018 and aimed to reduce anaemia prevalence in children, pregnant women and women of reproductive age by six interventions: (1) Periodic deworming, (2) Iron and folic acid supplementation, (3) Testing and treatment of anaemia in field settings, (4) Foods fortified with iron, folic acid and vitamin B12, (5) Behavioural Change Communication Campaign and (6) The screening and treatment of non-nutritional anaemia.^9^ Anaemia is often considered a proxy for iron deficiency (ID), with an estimated 50% of anaemia cases believed to be the result of ID.^4^ However, data gaps exist with regard to the micronutrient causes of anaemia, and the extent to which different micronutrients contribute to anaemia varies globally and even within countries.^10^ The underlying micronutrient cause(s) of anaemia can be determined using the mean corpuscular volume (MCV) of red blood cells and can be categorised as microcytic (MCV <80 femtolitres (fL), normocytic (MCV 80–100 fL) and macrocytic (MCV >100 fL).^8^ The MCV provides an insight into the potential micronutrient(s) responsible for an individual’s anaemia since it is widely accepted that microcytic and, to a lesser extent, normocytic anaemia may be caused by ID, while deficiencies in vitamin B12 and folate would result in macrocytic anaemia.^11^ Nevertheless, many studies conducted in low-income and middle-income countries use point-of-care methods such as Sahli’s method^12^ or the HemoCue device (HemoCue, Angelholm, Sweden) to estimate haemoglobin levels to determine anaemia status, due to the logistical difficulties and cost associated with the gold-standard laboratory-based auto-analyser methods.^13^,^17^ Although such point-of-care methods provide rapid results and are less invasive than the gold-standard methods, they are widely deemed to be more subjective and less precise, so estimated haemoglobin levels should be interpreted with caution.^18 19^ In addition, point-of-care tests do not allow the MCV to be determined, thus failing to provide information on the potential micronutrient cause(s) of anaemia.

This highlights a need for objective data which provides true estimates of haemoglobin levels, micronutrient deficiencies, and therefore the underlying nutritional causes of anaemia. Likewise, the use of birth cohorts provides an opportunity to study the life course and intergenerational effects of anaemia, as well as epigenetic factors.^20^

Although a number of birth cohorts have been established in India, the primary focus of such cohorts has been the identification of metabolic and vascular risk factors associated primarily with cardiovascular disease (CVD) and type 2 diabetes.^21^,^23^ Thus, there is a gap for a birth cohort study which focuses primarily on the life-course causes and intra/inter-generational consequences of anaemia, that is, from preconception, during pregnancy and in the children.

The Maharashtra Anaemia Study 3 (MAS 3) is nested in the Pune maternal nutrition study (PMNS), a longitudinal research project designed and implemented by the Diabetes Unit of the King Edward Memorial Hospital Research Centre, Pune, India. Findings from PMNS have been extensive, with the most notable being the first reported association in Indian children between LBW and future diabetes risk factors.^24^ The PMNS study is ongoing, with the most recent follow-up visit being conducted when children are turning 24 years of age. This paper introduces the MAS 3 cohort, which consists of data collected from the participants in the PMNS study from the antenatal period to children at 18 years of age (1996–2014). The MAS 3 cohort aims (1) To investigate nutritional, environmental and economic impacts on haemoglobin concentration/anaemia, (2) To identify the underlying micronutrient causes of anaemia and (3) To investigate the associations between anaemia and physical and cognitive development of Indian children during their first 18 years of life. The cohort includes data across a variety of timepoints and contains data on anthropometry, medical history, obstetric history (mothers only), SES, dietary intake, hygiene and comprehensive blood investigations, including nutritional biomarkers. Data available from the MAS 3 cohort will allow key objectives to be addressed:

- Longitudinal trends of haemoglobin concentration and micronutrients that are relevant to anaemia (iron, ferritin, folate, vitamin B12) from ages 6 to 18.
- Intergenerational and intragenerational risk factors associated with anaemia, and impaired physical and cognitive development.
- Nutritional, economic and environmental impacts on haemoglobin concentration/anaemia, physical and cognitive development.

## MAS 3 cohort description

**Figure 1:**
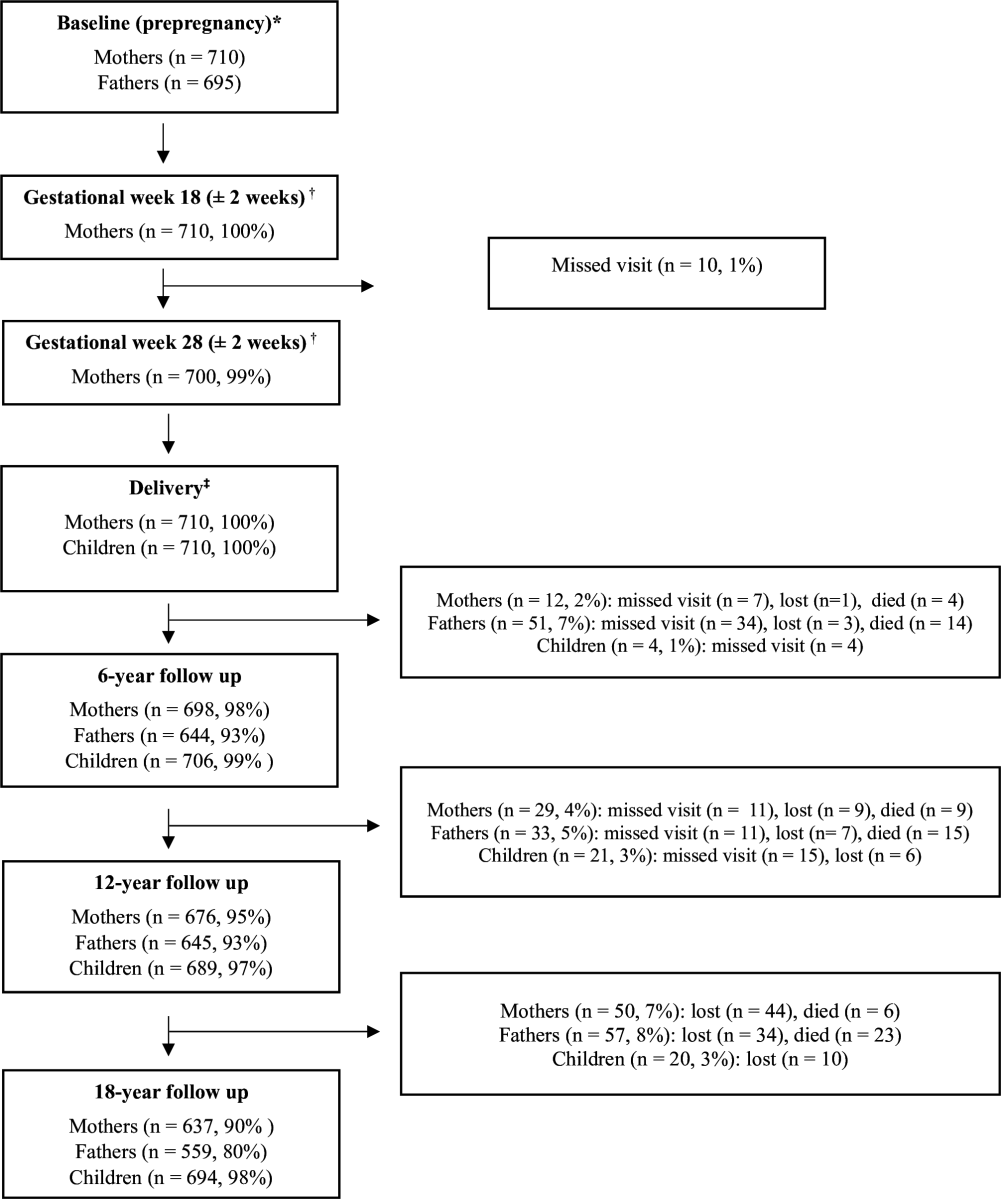
Participant flowchart of mothers, fathers and children at each timepoint within Maharashtra Anaemia Study 3. *Baseline visit for mothers and fathers was the prepregnancy visit. ^†^Mothers only: pregnancy visits at 18 weeks and 28 weeks of gestation. ^‡^Children’s baseline visit. Collected within 72 hours of delivery. Missed visit refers to participants who did not attend a given follow-up visit but did attend a future follow-up visit. Lost refers to participants who were considered lost to follow-up as they did not attend a given follow-up visit or any future visits. Reasons for missed visits and lost to follow-up are unknown. Percentages reported indicate the proportion of participants who attended a given visit relative to the number of participants at baseline.

The MAS 3 is designed from the PMNS, which is a prospective longitudinal maternal-child (birth) cohort established near Pune city in the Maharashtra state of India. The MAS 3 cohort is nested in the PMNS and did not include any additional data collection from participants, but MAS 3 differs from PMNS in its aims and objectives and the additional investigations of stored biological samples. The MAS 3 cohort uses data collected from PMNS, which took place in six villages, within approximately 50 km of Pune City. The study was approved by the KEMHRC Ethics Committee, and the village community leaders agreed to the study being conducted. Due to the MAS 3 cohort being nested in the PMNS, the recruitment strategy and study design of MAS 3 are synonymous with those of PMNS. As such, PMNS aimed to recruit all married non-pregnant women of childbearing age (15–40 years), and their husbands, living within the study area between June 1994 and April 1996.^25^,^32^ Of the 2466 married women recruited to PMNS during June 1994 and April 1996, 1102 became pregnant during the study period, and 710 of the children (and their parents) were included in the MAS 3 cohort (figure 1). The recruitment strategy for PMNS consisted of house-to-house surveys to identify eligible married couples; couples were excluded from recruitment if either they or their partner had sterilisation surgery. Those eligible consented to participate in the study by signing a written consent form. Illiterate participants consented with a fingerprint impression, following a verbal explanation of the study. Following entry into the study, women were visited monthly by field workers who recorded the date of their last menstrual period (LMP). Those with two successive missed menstrual periods had an ultrasound examination confirming pregnancy. LMP was used to calculate gestational age, except in cases where it differed from the ultrasound estimate by more than 2 weeks, for which the latter was used. The women’s (and father’s) baseline visit was the final visit before two successive missed menstrual periods were reported. Field visits were coordinated using a computerised database listing the women’s names and locations, dates of their LMP and field visits. To aid with participant engagement, a concerted effort was made by the research team to build close rapport with each of the communities involved in the study. In addition, the long-term follow-up visits (when children were aged 6 years, 12 years and 18 years) were conducted at the KEMHRC in Pune city. Participants were provided with accommodation and meals, as well as reimbursement for travel to the research centre.

### MAS 3 inclusion criteria

#### Women

- Married, non-pregnant (at the time of recruitment).
- Those who went on to have a singleton pregnancy, which was identified <21 weeks’ gestation.
- Pregnancies which continued to full term and resulted in a live birth.

#### Children

- Children with follow-up data available for at least one of the long-term timepoints (6 years, 12 years and 18 years).

### MAS 3 exclusion criteria

#### Women

- Those who migrated out of the study area following recruitment and were unable to continue participation in the study.
- Withdrawal from the study during recruitment or follow-up phase.
- Those who did not have a singleton pregnancy.
- Lack of availability of data on pregnancy or delivery outcomes.
- Women and/or their partners had sterilisation surgery.

#### Children

- Children born with congenital anomalies or other medical conditions which affected their ability to continue their study participation.
- Children who did not attend at least one of the follow-up visits (6 years, 12 years and 18 years).

## MAS 3 cohort

Enrolment of the pregnant mothers and their husbands (F0 generation) occurred between June 1994 and April 1996, while the births of the F1 generation (children) occurred between November 1994 and September 1996. Women whose pregnancy met the eligibility criteria were followed up twice during their pregnancy: gestational week 18 (±2 weeks) and gestational week 28 (±2 weeks). At the time of delivery, data were collected from both the women and their children. Deliveries occurred at government Public Health Centres, subcentres located in the same six villages that the women were recruited from, as well as private hospitals and in-home settings. The final 18-year follow-up of the F1 generation was completed by January 2015.

### What has been measured?

**Figure 2:**
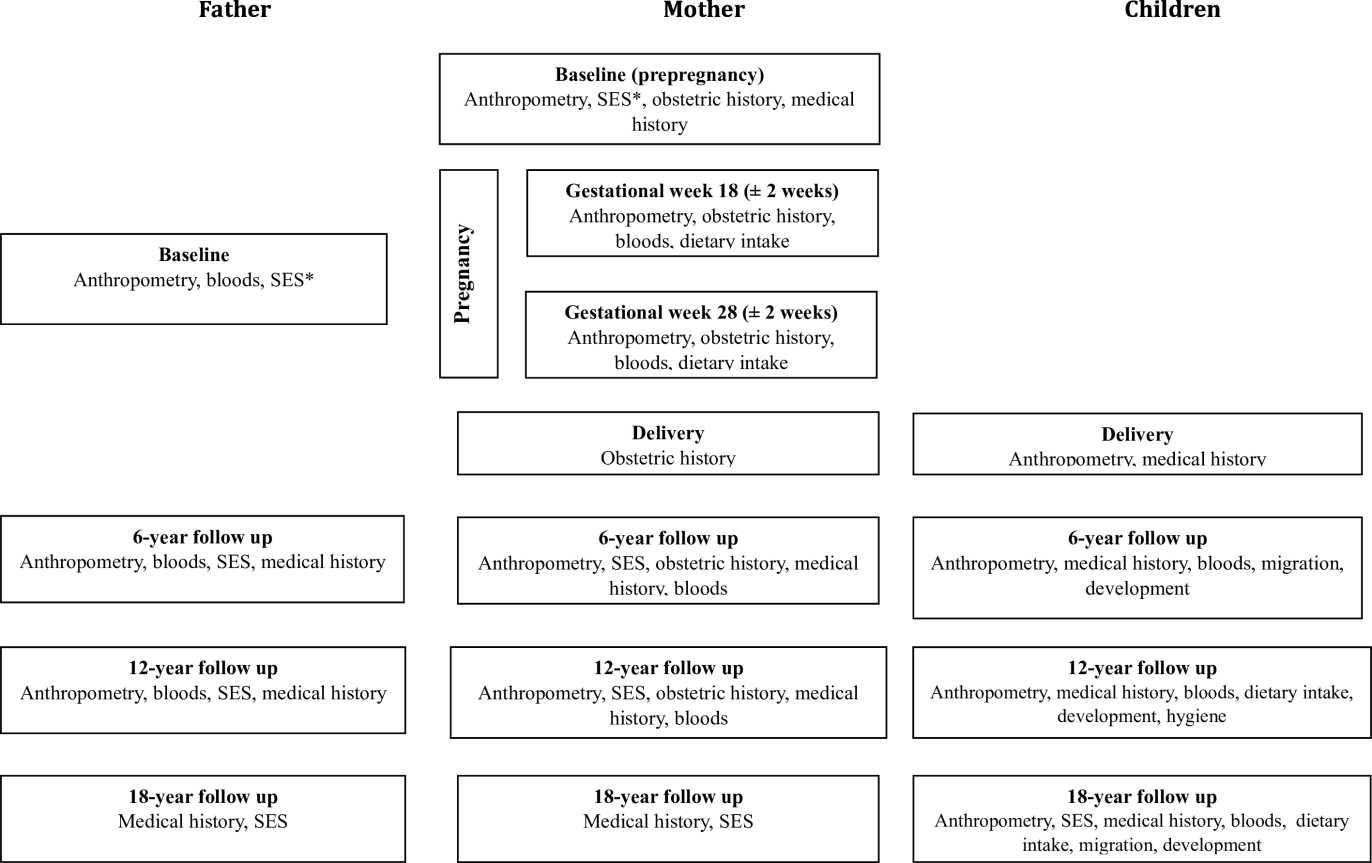
Flowchart of data collection available for Maharashtra Anaemia Study 3. *Household socioeconomic status.

Figure 2 provides an overview of the data collection at each timepoint, while table 1, online supplemental tables S1 and S2 provide additional detail for the data collected from children, mothers and fathers, respectively.

**Table 1:** Child data collected at delivery and long-term follow-up for Maharashtra Anaemia Study 3

	Delivery	Age 6	Age 12	Age 18
Anthropometry				
Birth weight, length, abdominal*, foot length, chest*, triceps, subscapular	✓			
Head*, MUAC	✓	✓	✓	✓
Height, weight, BMI, waist*, hip*, skinfold†		✓	✓	✓
Systolic and diastolic BP, pulse		✓	✓	✓
Medical history				
Malformations, complications	✓			
Fever, cough, cold, vomiting, diarrhoea (chronic and acute), jaundice, URI, LRI, ear discharge, night blindness, skin problems, asthma, measles, parasitic infections, immunisation history, sphincter control, scholastic performance, dental status, visual acuity, medical examination		✓		
Medical examination		✓	✓	✓
Menarche, history of chronic illness, vitamins/minerals, homeopathic drugs, pallor, hyperpigmentation, musculoskeletal deformities, allergies, acanthosis nigricans, neck grade, axilla grade, prescribed medications				✓
Smoking and alcohol consumption				✓
Bloods‡				
WBC, RBC, Hb, Haematocrit, MCV, MCH, MCHC, platelet count, lymphocyte§, monocyte§, granulocyte§, RDW, MPV, creatinine, vitamin B12, folate, CRP, ferritin, iron, H.pylori, albumin, total protein, OGTT		✓	✓	✓
Plateletcrit, PDW			✓	✓
Nutrition				
Breast feeding duration, weaning		✓		
Serving size, frequency consumed (weekly, daily, monthly, yearly), number of servings, seasonality, monthly seasonal frequency, daily quantity intake, daily calorie intake, daily carbohydrate intake, daily fat intake, daily protein, daily iron intake¶			✓	✓
Total daily calorie, proteins, fats, carbohydrates and iron intake**			✓	✓
Migration				
Place of migration, reason for migration, years of migration		✓		✓
Age at migration				✓
Development				
IQ test		✓		
Attending school, standard at school, CPM test, picture completion, digit span (forward and backwards), trail making, AVLT total learning score, block design score			✓	
Education				✓
Hygiene				
Questionnaire			✓	
SES				
Occupation				✓

* Circumference.

† Bicep, tricep, subscapular and suprailiac.

‡ An additional four blood investigations (complete blood count, folate, vitamin B12, glucose) are also available at age 17 years.

§ Count and percentage.

¶ Reported for the following food types: beverages, chapati/roti, rice, pulses, legumes, vegetables, GLV, chutneys, fasting foods, fruits, meat/fish, milk products, bakery items, spicy snacks, sweet snacks, festival foods and special foods.

** Derived by calculating the total daily intake across all food types reported.

AVLT, Auditory-Verbal Learning Test; BMI, body mass index; BP, blood pressure; CRP, C-reactive volume; H.pylori, Helicobacter pylori; IQ, intelligence quotient; LRI, lower respiratory infection; MCH, mean corpuscular haemoglobin; MCHC, Mean corpuscular haemoglobin concentration; MCV, mean corpuscular volume; MPV, mean platelet volume; MUAC, mid-upper arm circumference; OGTT, oral glucose tolerance test; PDW, platelet distribution width; RBC, red blood cells; RDW, red cell distribution width; SES, socioeconomic status; URI, upper respiratory infection; WBC, white blood cells.

### Anthropometry

The data collected at a given timepoint varied by participant (ie, mothers, fathers and children), yet the following data are available for all participants from at least one timepoint: height, weight, body mass index (BMI), hip circumference, waist circumference, head circumference, mid-upper arm circumference, blood pressure and pulse. Data collected on children within 72 hours post-delivery were birth weight, length, abdominal circumference, foot length, chest circumference, triceps circumference and subscapular circumference. Details of how child anthropometry was measured at birth have been previously reported.^26^

### Obstetric history

Information regarding mothers’ gravidity, parity, abortion history, number of living children and preterm delivery history was recorded during the first pregnancy visit (Gestational week 18), as well as the long-term follow-up (6-year and 12-year). Factors indicating mothers’ health were recorded at both pregnancy visits (Gestational weeks 18 and 28), which included vomiting, diarrhoea, jaundice, vaginal bleeding, current medications and consumption of iron tablets. Following delivery, intra-natal and antenatal complications, mode and outcome of delivery, and postpartum outcomes were recorded.

### Medical history

For mothers and fathers, the medical history focused on existing diagnosed conditions, including diabetes mellitus, hypertension, tuberculosis, ischaemic heart diseases, chronic renal diseases, anaemia, asthma and stroke (online supplemental tables S1 and S2). At the baseline visit, mothers provided a comprehensive overview of their medical history, while a more basic medical history was collected from fathers at varying timepoints. For children, malformations and complications were recorded following birth and a comprehensive list of conditions common to young children was collected when children were 6 years of age. Children underwent a medical examination at each of the long-term follow-up visits (age 6, age 12, age 18). Children also provided information aimed at gaining an insight into their physical development, as well as information on their smoking and alcohol consumption at ages 12 and 18, respectively.

### Blood tests

Participants had undergone comprehensive blood investigations (online supplemental table S3), which have been reported previously.^28 33^ The following blood investigations are available for all children at each of the longitudinal follow-ups in MAS 3 (age 6, age 12, age 18): albumin, complete blood count (CBC), creatinine, ferritin, *Helicobacter pylori* antibodies, highly sensitive C-reactive protein (hS-CRP), oral glucose tolerance test (OGTT) total protein, and serum/plasma vitamin B12. An additional four blood investigations (CBC, folate, vitamin B12, glucose) are also available at age 17 years. In contrast, mothers and fathers have data available for ferritin, folate, vitamin B12, CBC and OGTT. For mothers, blood samples were collected at gestational week 18 (±2 weeks) and gestational week 28 (±2 weeks), while blood samples were collected from fathers at either of the pregnancy visits (gestational week 18 or week 28), depending on the father’s availability.

### Dietary intake

The 24-hour dietary recall approach was used and was modified to include information on portion sizes and was collected from mothers at each of the pregnancy visits (gestational weeks 18 and 28).^33^ The 24-hour dietary recall was collected via an interview of the participant by a nutritionist, and consumed food items were recorded in chronological order from morning until evening meal. The nutritionists ensured the participants were not unwell or fasting at the time of the interview, and that foods consumed outside of the home were also recorded. A food-frequency questionnaire (FFQ) was also administered to obtain data on the frequency of consumption of 17 food categories over the preceding 3 months. The 17 food categories included: pulses, legumes, vegetables, green leafy vegetables, chapati/roti, rice, meat/fish, milk products, chutneys, fasting foods, fruits, bakery items, sweet snacks, spicy snacks, festival foods, special foods and beverages. The food categories were mutually exclusive, and participants could indicate consumption with eight frequency options, which ranged from ‘never’ to ‘more than once daily’. Data on the duration of breastfeeding and whether milk was used during weaning were collected retrospectively at the 6-year follow-up. To create the nutritional values of foods commonly consumed by the cohort, 288 distinct food preparations were analysed in the laboratory to obtain estimates of their protein, fat and carbohydrate content, as described previously.^33^ The micro-Kjeldahl method, using the 1030 Kjeltec Autoanalyser (Tecator AB, Höganäs, Sweden), was used on dried samples to estimate protein content, the Soxhlet method for fat,^34^ and carbohydrates were estimated by subtraction. For children, information relating to foods consumed and the frequency of consumption was recorded using a semi-quantitative FFQ at the 12-year and 18-year follow-up. At the 12-year follow-up, the FFQ collected data on 99 different food items belonging to 13 different food categories. A nutrition transition survey was also used to collect data on the frequency at which participants consumed food outside of the home and included 24 food items under six different categories. At the 18-year follow-up, an FFQ was used to collect data on 115 different food items in 15 categories. A nutrition transition questionnaire was also used, but this time data were collected on 31 food items in seven different categories. For children, the FFQ and nutrition transition questionnaires were used to collect data in the 6 months preceding the scheduled follow-up visit. From the FFQ, monthly scores for each food item were calculated and combined with scores from food items in the same category. The aggregated scores were then categorised into eight frequency options, on an ordinal scale which ranges from ‘never’ to ‘more than once daily’.

### Migration

Data on the number of times the children had migrated, that is, moved from their village of birth, reasons for migration, the years between each subsequent migration and the age of the child when the migration occurred were obtained via a questionnaire. Data were collected at the 6-year and 12-year follow-up.

### Hygiene

A hygiene questionnaire was completed at the 12-year follow-up by the child’s parent or guardian, or field worker if the parents were illiterate. The questionnaire focused on the occurrence of hand washing after defecation and before eating, along with the substance (such as soap) used to wash hands.

### Socioeconomic status

The SES for each household was obtained at the prepregnancy visit using a standardised questionnaire, developed for rural populations, which scored individuals using indicators reflective of the cohort population at that time.^35^ The questionnaire scores individuals on nine characteristics: caste of family, occupation of head of household, household family type that is, single or joint, including extended family, and size, land possession indicated by acreage/size, type of house, household material possessions, farm power, assessed by number of animals, social participation in a local organisation and education level of the head of household.^36^ At each of the long-term follow-ups, however, SES was reported using the India national family health survey standard of living index (SLI).^37^ The SLI scores individuals using a total of 27 questions that cover house type, toilet facility, source of lighting that is, kerosene, gas or oil, or electricity, main fuel for cooking, source of drinking water, separate room for cooking, home ownership, ownership of agricultural land, ownership of irrigated land, ownership of livestock and ownership of durable goods.^37^ Total scores are classified into low (0–14), medium (15–24) and high categories (25–67).^37^ Data relating to parental education and occupation were also collected. At the parental baseline (prepregnancy) visit and 6-year follow-up, education in years were reported for both mothers and fathers. At the 12-year and 18-year follow-up visits, the occupation of mothers and fathers was collected.

### Patient and public involvement

There was no patient or public involvement in this study.

## Findings to date

### MAS 3 cohort characteristics (n=710)

This cohort profile is the first publication on the MAS 3 cohort study, as analyses are ongoing. At the time of recruitment, the mean age of mothers was 21 years (range 15–38 years) and a third (31%) had received between six and eight years of education, while 23% reported not having received any form of education (table 2). Most women were underweight (65.0% defined as BMI <18.5 kg/m^2^), mean weight was 41.7 kg (SD 5.0), while 56.0% of their husbands had their BMI within the WHO defined normal range of 18.5–24.9 kg/m^2^ (online supplemental table S4). A third (31%) of the women had been pregnant at least once before, while 29.0% were primigravida. Over half of the children born were boys (52.0%), the majority (69.0%) had a birth weight >2500 g, with approximately a third (31.0%) of LBW (birth weight <2500 g^38^). The mean length of the children was 47.5 cm (range 30.8–56.0 cm), the mean head circumference was 32.9 cm (range 27.0–36.2) and the mean subscapular skinfold was 4.2 mm (range 2.4–9.5) (table 2). Most children were born into households, which included more than five adults (65%), and the head of the household had received up to a primary level of education (45%) (online supplemental table S4).

**Table 2:** Baseline cohort characteristics (mothers and children)

Variables	Total sample (N)	Mean (SD)/n (%)
**Mothers (baseline)** *		**n (%)**
Age	708	
Under 18		68 (9.6%)
18–25		517 (73%)
26–30		100 (14%)
31–38		23 (3.2%)
Height (cm)	710	151.96 (4.93%)
Weight (kg)	704	41.68 (4.97%)
BMI (kg/m^2^)^†^	704	
Underweight		455 (65%)
Normal		246 (35%)
Overweight		2 (0.3%)
Obese		1 (0.1%)
Education (years)	688	
0 years		160 (23%)
1–5 years		131 (19%)
6–8 years		212 (31%)
9–11 years		150 (22%)
12–13 years		24 (3.5%)
≥14 years		11 (1.6%)
Gravida	708	
1		208 (29%)
2		222 (31%)
3		175 (25%)
>4		103 (15%)
Parity	708	
0		226 (32%)
1		245 (35%)
2		157 (22%)
3		50 (7.1%)
>4		30 (4.2%)
Abortion^‡^	708	
0		637 (90%)
≥1		71 (10%)
MTP^‡^	708	
0		694 (98%)
≥1		14 (2.0%)
Still birth^‡^	708	
0		695 (98.2%)
1 to 2		13 (1.8%)
Neonatal death^‡^	708	
0		689 (97.3%)
≥1		19 (2.7%)
Preterm delivery^‡^	708	
0		695 (98.2%)
≥1		13 (1.8%)
**Children (baseline)** *		**Mean (SD)/n (%)**
Sex	710	
Male		370 (52%)
Female		340 (48%)
Birth weight (g)	668	2629.63 (375.93%)
LBW (<2500 g)^§^	668	206 (31%)
VLBW (<1500 g)^§^	668	1 (0.1%)
Length (cm)	698	47.49 (2.27%)
Abdominal circumference (cm)	698	28.47 (2.05%)
Head circumference (cm)	698	32.90 (1.44%)
Chest circumference (cm)	698	30.98 (1.85%)
Mid-upper arm circumference (cm)	698	9.59 (0.95%)
Triceps (mm)	698	4.21 (0.86%)
Subscapular (mm)	698	4.18 (0.88%)
Foot length (cm)	697	7.76 (0.52%)

* Baseline visit for mothers is the final prepregnancy visit. Baseline visit for children occurred within 72 hours of birth.

† Calculated using WHO BMI categories: underweight (BMI <18 kg/m2); normal (BMI ≥18.5 kg/m2); overweight (BMI ≥25 kg/m2); obese (BMI ≥30 kg/m2).

‡ Recorded at the baseline visit as part of the mother’s obstetric history and refers to the occurrence of such events in previous pregnancies, if applicable.

§ Defined according to WHO definitions.

BMI, body mass index; LBW, low birth weight; MTP, medically terminated pregnancy; VLBW, very low birth weight.

### Findings from PMNS

Findings from PMNS, however, have been widely published.^2527 29 31 33 39^,^44^ Unlike MAS 3, the aims of the PMNS study were to study determinants of fetal growth and to document life course evolution of cardiometabolic risk factors and their early life determinants.^39^ A number of risk factors have been suggested as contributing to adverse fetal growth. Maternal folate status and micronutrient-rich foods, maternal vitamin B12 deficiency, represented by plasma total homocysteine (tHcy) concentration and maternal folate and plasma glucose, have been reported as associated with child birth size.^31 33 40^ In addition, maternal inflammation, seasonal differences in maternal activity levels and food intake, and excessive maternal activity during pregnancy have also been suggested to adversely affect child birth size.^29 41 42^ While maternal anthropometry, as well as paternal anthropometry, is believed to adversely affect a number of child growth indicators.^25^ In addition, results from PMNS have identified potential risk factors for CVD outcomes in children during childhood and early adulthood. Maternal nutritional intake and circulating concentrations of methylmalonic acid, tHcy, folate and vitamin B12 during pregnancy were found to be correlated with body composition and insulin resistance at age 6.^27^ Likewise, child body anthropometry measures at birth and rapid growth were found to be associated with increased glucose concentrations, blood pressure and relative insulin resistance, respectively, at age 6.^43^

## Strengths and limitations

MAS 3 has many strengths. Compared with other prospective birth cohorts in India, which also focus on maternal anaemia and child outcomes,^14^,^1745^ the MAS 3 cohort’s follow-up period of 18 years (following birth) is the only cohort known to the authors which allows the study of the life-course causes and intra/inter-generational consequences of anaemia. Many such cohorts in India have short follow-up periods of <3 years after birth,^14^,^1745^ thus preventing information on whether associations and risk factors identified during the first 2 years of life continue through childhood and into adulthood. In addition, unlike the MAS 3 cohort, other anaemia-related birth cohorts in India do not include more than one follow-up visit and are unable to investigate longitudinal trends in haemoglobin concentrations and related micronutrients (iron, ferritin, folate and vitamin B12).^14^,^1745^ Few anaemia-related birth cohorts have been conducted in other South Asian countries.^57^,^59^ These cohorts have sample sizes ranging from 629–1584. Although two of the three cohorts have a larger sample size compared with the MAS 3 cohort.^58 59^ All three South Asian cohorts have substantially shorter follow-up periods, which end at delivery, did not include data collection on the children’s father, did not include extensive blood investigations, which could enable the underlying micronutrient causes of anaemia (if applicable) to be determined, and did not report whether discrepancies over gestational age were resolved using ultrasound examination. An additional strength of the MAS 3 cohort is the high retention of participants across the long-term follow-up, which is >90% for mothers and children, and ≥80% for fathers (figure 1). Such a high response rate was likely influenced by the close rapport developed and maintained with the local communities by the research team based at the Diabetes Unit of the KEMHRC. There are also some limitations that must be considered. Although this is a population-based study, the population cannot be considered representative of Maharashtra state or India as a whole. Recruitment occurred in six villages, within 50 km around Pune city, which means results from this study are unlikely to be generalisable to urban or more affluent areas across the state or country. However, the results may be representative of other rural settings where the population is predominantly of lower or middle socioeconomic groups and belongs to farming families in a rural Indian set-up. Although a strength of this study is the use of highly trained field workers for data collection, the risk of interviewer bias should be considered since all questionnaires were completed by field workers as participants were questioned. This was, however, unavoidable due to the lack of formal education for many within the study population and the need for handwritten data collection forms in the study.

## Collaboration

The authors welcome and encourage research collaboration with other researchers. Those interested are invited to contact the corresponding authors.

## Supplementary material

10.1136/bmjopen-2025-104184 online supplemental file 1

## Data Availability

Data may be obtained from a third party and are not publicly available.

## References

[1] Wedderburn CJ, Ringshaw JE, Donald KA (2022). Association of Maternal and Child Anemia With Brain Structure in Early Life in South Africa. JAMA Netw Open.

[2] International Institute for Population Sciences (IIPS) and ICF (2021). National Family Health Survey (NFHS-5), 2019-21.

[3] Victora CG, Christian P, Vidaletti LP (2021). Revisiting maternal and child undernutrition in low-income and middle-income countries: variable progress towards an unfinished agenda. Lancet.

[4] Singh SK, Lhungdim H, Shekhar C (2023). Key drivers of reversal of trend in childhood anaemia in India: evidence from Indian demographic and health surveys, 2016-21. BMC Public Health.

[5] Young MF, Oaks BM, Tandon S (2019). Maternal hemoglobin concentrations across pregnancy and maternal and child health: a systematic review and meta-analysis. Ann N Y Acad Sci.

[6] Ministry of Health and Family Welfare (MoHFW), Government of India (2023). Comprehensive national nutrition survey (2016-2018). https://knowledge.unicef.org/resource/comprehensive-national-nutrition-survey-2016-2018.

[7] Ministry of Health and Family Welfare, Government of India (2023). District level household survey (DLHS). https://www.iipsdata.ac.in/major_studies/4/project_details.

[8] Kumar SB, Arnipalli SR, Mehta P (2022). Iron Deficiency Anemia: Efficacy and Limitations of Nutritional and Comprehensive Mitigation Strategies. Nutrients.

[9] National Health Mission Department of Health & Family Welfare, Ministry of Health & Family Welfare, Government of India. National Health Mission Anaemia Mukt Bharat. https://nhm.gov.in/index1.php?lang=1&level=3&sublinkid=1448&lid=797.

[10] Venkatesh U, Sharma A, Ananthan VA (2021). Micronutrient’s deficiency in India: a systematic review and meta-analysis. J Nutr Sci.

[11] Wang M (2016). Iron Deficiency and Other Types of Anemia in Infants and Children. Am Fam Physician.

[12] Ahankari AS, Dixit JV, Fogarty AW (2016). Comparison of the NBM 200 non-invasive haemoglobin sensor with Sahli’s haemometer among adolescent girls in rural India. BMJ Innov.

[13] Yadav K, Kant S, Ramaswamy G (2020). Validation of Point of Care Hemoglobin Estimation Among Pregnant Women Using Digital Hemoglobinometers (HemoCue 301 and HemoCue 201+) as Compared with Auto-Analyzer. Indian J Hematol Blood Transfus.

[14] Patel A, Prakash AA, Das PK (2018). Maternal anemia and underweight as determinants of pregnancy outcomes: cohort study in eastern rural Maharashtra, India. BMJ Open.

[15] Heesemann E, Mähler C, Subramanyam MA (2021). Pregnancy anaemia, child health and development: a cohort study in rural India. BMJ Open.

[16] Jessani S, Saleem S, Hoffman MK (2021). Association of haemoglobin levels in the first trimester and at 26–30 weeks with fetal and neonatal outcomes: a secondary analysis of the Global Network for Women’s and Children’s Health’s ASPIRIN Trial. BJOG.

[17] Shobeiri F, Begum K, Nazari M (2006). A prospective study of maternal hemoglobin status of Indian women during pregnancy and pregnancy outcome. Nutr Res.

[18] Abraham RA, Agrawal PK, Johnston R (2020). Comparison of hemoglobin concentrations measured by HemoCue and a hematology analyzer in Indian children and adolescents 1-19 years of age. Int J Lab Hematol.

[19] Hinnouho G-M, Barffour MA, Wessells KR (2018). Comparison of haemoglobin assessments by HemoCue and two automated haematology analysers in young Laotian children. J Clin Pathol.

[20] Lawlor DA, Andersen A-M, Batty GD (2009). Birth cohort studies: past, present and future. Int J Epidemiol.

[21] Krishna M, Kalyanaraman K, Veena SR (2015). Cohort Profile: The 1934-66 Mysore Birth Records Cohort in South India. Int J Epidemiol.

[22] Huffman MD, Khalil A, Osmond C (2015). Association between anthropometry, cardiometabolic risk factors, & early life factors & adult measures of endothelial function: Results from the New Delhi Birth Cohort. Indian J Med Res.

[23] Antonisamy B, Raghupathy P, Christopher S (2009). Cohort Profile: the 1969-73 Vellore birth cohort study in South India. Int J Epidemiol.

[24] Yajnik CS, Fall CH, Vaidya U (1995). Fetal growth and glucose and insulin metabolism in four-year-old Indian children. Diabet Med.

[25] Wills AK, Chinchwadkar MC, Joglekar CV (2010). Maternal and paternal height and BMI and patterns of fetal growth: the Pune Maternal Nutrition Study. Early Hum Dev.

[26] Yajnik CS, Fall CHD, Coyaji KJ (2003). Neonatal anthropometry: the thin–fat Indian baby. The Pune Maternal Nutrition Study. Int J Obes.

[27] Yajnik CS, Deshpande SS, Jackson AA (2008). Vitamin B12 and folate concentrations during pregnancy and insulin resistance in the offspring: the Pune Maternal Nutrition Study. Diabetologia.

[28] Joshi NP, Kulkarni SR, Yajnik CS (2005). Increasing maternal parity predicts neonatal adiposity: Pune Maternal Nutrition Study. Am J Obstet Gynecol.

[29] Rao S, Kanade AN, Yajnik CS (2009). Seasonality in maternal intake and activity influence offspring’s birth size among rural Indian mothers--Pune Maternal Nutrition Study. Int J Epidemiol.

[30] Kinare AS, Chinchwadkar MC, Natekar AS (2010). Patterns of fetal growth in a rural Indian cohort and comparison with a Western European population: data from the Pune maternal nutrition study. J Ultrasound Med.

[31] Kulkarni SR, Kumaran K, Rao SR (2013). Maternal Lipids Are as Important as Glucose for Fetal Growth. Diabetes Care.

[32] Katre P, Yajnik CS (2015). Influence of early life environment on risk of non-nommunicable diseases (NCDs) in Indians.

[33] Rao S, Yajnik CS, Kanade A (2001). Intake of micronutrient-rich foods in rural Indian mothers is associated with the size of their babies at birth: Pune Maternal Nutrition Study. J Nutr.

[34] Raghuramulu N, Madhavan K, Kalyanasundaram S (1983). A manual of laboratory techniques.

[35] Pareek U, Trivedi G (1964). Reliability and validity of socio-economic scales. Indian J Appl Psychol.

[36] Kishore J, Kohli C, Kumar N (2017). Scales used in India to evaluate socioeconomic status in Medical Research: limitations and need of review for more comprehensive scale. J Int Med Sci Acad.

[37] International Institute for Population Sciences (1998). International institute for population sciences and ORC Macro 2001 national family health survey (NFHS-2).

[38] Department of Nutrition for Health & Development, World Health Organization (2014). Low birth weight policy brief.

[39] MRC Lifecourse Epidemiology Centre The Pune Maternal Nutrition Study. https://www.mrc.soton.ac.uk/cohorts/mrclec-collaborations-in-india/the-pune-maternal-nutrition-study/.

[40] Yajnik CS, Deshpande SS, Panchanadikar AV (2005). Maternal total homocysteine concentration and neonatal size in India. Asia Pac J Clin Nutr.

[41] Rao S, Kanade A, Margetts BM (2003). Maternal activity in relation to birth size in rural India. The Pune Maternal Nutrition Study. Eur J Clin Nutr.

[42] Bhat DS (2011). Maternal c-reactive protein is a predictor of neonatal size at birth: Pune Maternal Nutrition Study.

[43] Joglekar CV, Fall CHD, Deshpande VU (2007). Newborn size, infant and childhood growth, and body composition and cardiovascular disease risk factors at the age of 6 years: the Pune Maternal Nutrition Study. Int J Obes.

[44] Yajnik CS, Katre PA, Joshi SM (2015). Higher glucose, insulin and insulin resistance (HOMA-IR) in childhood predict adverse cardiovascular risk in early adulthood: the Pune Children’s Study. Diabetologia.

[45] Hirve SS, Ganatra BR (1994). Determinants of low birth weight: a community based prospective cohort study. Indian Pediatr.

[46] Malhotra M, Sharma JB, Batra S (2002). Maternal and perinatal outcome in varying degrees of anemia. Int J Gynaecol Obstet.

[47] Kumar A, Chaudhary K, Prasad S (2010). Maternal indicators and obstetric outcome in the north Indian population: a hospital-based study. J Postgrad Med.

[48] Finkelstein JL, Kurpad AV, Bose B (2020). Anaemia and iron deficiency in pregnancy and adverse perinatal outcomes in Southern India. Eur J Clin Nutr.

[49] Metgud CS, Naik VA, Mallapur MD (2012). Factors affecting birth weight of a newborn--a community based study in rural Karnataka, India. PLoS ONE.

[50] Bora R, Sable C, Wolfson J (2014). Prevalence of anemia in pregnant women and its effect on neonatal outcomes in Northeast India. J Matern Fetal Neonatal Med.

[51] Kattula D, Sarkar R, Sivarathinaswamy P (2014). The first 1000 days of life: prenatal and postnatal risk factors for morbidity and growth in a birth cohort in southern India. BMJ Open.

[52] Menon KC, Ferguson EL, Thomson CD (2016). Effects of anemia at different stages of gestation on infant outcomes. Nutrition.

[53] Ahankari AS, Myles PR, Dixit JV (2017). Risk factors for maternal anaemia and low birth weight in pregnant women living in rural India: a prospective cohort study. Public Health (Fairfax).

[54] Salunkhe AH, Pratinidhi A, Sv K (2018). CORRELATION OF NUTRITIONAL STATUS OF MOTHER AND THE BIRTH WEIGHT OF THE BABY. Asian J Pharm Clin Res.

[55] Salunkhe AH, Pratinidhi AK, Salunkhe JA (2019). Antenatal Risk Scoring Scale for Predication of Low Birth Weight and Its Validity. Indian J Community Med.

[56] Agarwal S, Agarwal A, Bansal AK (2002). Birth weight patterns in rural undernourished pregnant women. Indian Pediatr.

[57] Lone FW, Qureshi RN, Emanuel F (2004). Maternal anaemia and its impact on perinatal outcome. Trop Med Int Health.

[58] Abeysena C, Jayawardana P, Seneviratne RdA (2010). Maternal haemoglobin level at booking visit and its effect on adverse pregnancy outcome. Aust NZ J Obst Gynaeco.

[59] Mamun AA, Padmadas SS, Khatun M (2006). Maternal health during pregnancy and perinatal mortality in Bangladesh: evidence from a large-scale community-based clinical trial. Paediatr Perinat Epidemiol.

[60] Kumaran K, Yajnik P, Lubree H (2017). The Pune Rural Intervention in Young Adolescents (PRIYA) study: design and methods of a randomised controlled trial. BMC Nutr.

