# Supplementary material for: Cohort profile: the Maharashtra Anaemia Study 3 (MAS 3)—a maternal-child cohort study up to age 18 years in India
Source: BMJ Open. 2025 Oct 28;15(10):e104184. doi: 10.1136/bmjopen-2025-104184 (PMC12570902; doi:10.1136/bmjopen-2025-104184)
Supplement: online supplemental file 1 [file bmjopen-15-10-s001.docx]

**SUPPLEMENTARY MATERIAL**

**Table S1:** Maternal data collected (all visits) available for MAS 3

**Table S2:** Paternal data (all visits) available for MAS 3

**Table S3:** Laboratory investigations available for MAS 3

**Table S4:** Baseline cohort characteristics (fathers and household)

MAS 3 Project- Acknowledgments and contributions

| **Table S1:** Maternal data collected (all visits) available for MAS 3 | | | | | | |
| --- | --- | --- | --- | --- | --- | --- |
|  | Pre-pregnancy | Pregnancy | | Long-term follow-up visit | | |
|  | Baseline | Gestational week 18 | Gestational week 28 | 6 years | 12 years | 18 years |
| **Anthropometry** |  |  |  |  |  |  |
| Height, weight, BMI, waist^1^, hip^1^, arm^1^ | ✓ | ✓ | ✓ | ✓ | ✓* |  |
| Blood pressure, pulse |  | ✓ | ✓ | ✓ | ✓ |  |
| **Medical history** |  |  |  |  |  |  |
| Diabetes, hypertension, , IHD, | ✓ |  |  | ✓^‡^ |  | ✓ |
| Tuberculosis, chronic renal disease, anaemia, smoking and alcohol consumption, history of chewing betel nut | ✓ |  |  |  |  |  |
| Date and cause of death (if applicable) |  |  |  | ✓ |  | ✓ |
| Medical examination |  |  |  |  | ✓ |  |
| **Obstetric history** |  |  |  |  |  |  |
| Pregnancy status |  |  |  | ✓ | ✓ |  |
| Gravida, parity, abortion, |  | ✓ |  |  | ✓ |  |
| MTP, stillbirth, neonatal death, preterm delivery |  | ✓ |  |  |  |  |
| Vomiting, diarrhoea, fever, jaundice, vaginal bleeding, medication, iron tablets |  | ✓ | ✓ |  |  |  |
| **Bloods** |  |  |  |  |  |  |
| Hb, WBC, RBC, lymphocyte^2^, platelet count, haematocrit, vitamin B12, red cell folate, ferritin, OGTT |  | ✓ | ✓ | ✓^¶^ | ✓^¶^ |  |
| MCV, MCH, MCHC, monocytes^2^, granulocytes^2^, MPV, Plateletcrit^3^, PDW^3^ |  |  |  | ✓ | ✓ |  |
| **Nutrition** |  |  |  |  |  |  |
| 24-hour dietary recall |  | ✓ | ✓ |  |  |  |
| Food-frequency questionnaire |  | ✓ | ✓ |  |  |  |
| **SES status**^§^ |  |  |  |  |  |  |
| Caste of family, occupation of head of household, household family, land possession indicated by acreage, type of house, household material possessions, farm power, social participation in a local organisation, and education level of the head of household | ✓ |  |  |  |  |  |
| BMI: body mass index; IHD: ischaemic heart disease; MTP: medical termination of pregnancy; Hb: haemoglobin; WBC: white blood cell; RBC: red blood cell; OGTT: oral glucose tolerance test; MCV: mean corpuscular volume; MCH: mean corpuscular haemoglobin; MCHC: mean corpuscular haemoglobin count; MPV: mean platelet volume; PDW; platelet distribution width  SES: socioeconomic status  ^1^circumference  ^2^Number and percentage  ^3^ Collected at 12-year follow-up only  *Waist, hip and arm circumference not collected  ^‡^Medical history relating to asthma reported at 6-year follow-up.  ^¶^Fasting glucose, vitamin B12, red cell folate, and ferritin were not collected  ^§^Household socio-economic status obtained using the questionnaire by Pareek, U et al. (1964). The nine components of the questionnaire each consist of graded categories. The final composite score is the sum of the graded categories selected for that individual. | | | | | | |

**Table S2:** Paternal data (all visits) available for MAS 3

|  |  | Long-term follow-up visit | | |
| --- | --- | --- | --- | --- |
|  | Baseline | 6 years | 12 years | 18 years |
| **Anthropometry** |  |  |  |  |
| Height, weight, BMI | ✓ | ✓ | ✓ |  |
| Waist^1^, hip^1^, head^1^ | ✓ | ✓ |  |  |
| MUAC, skinfold (biceps, triceps, subscapular, suprailiac) |  | ✓ |  |  |
| Systolic BP, diastolic BP, pulse |  | ✓ | ✓ |  |
| **Medical history** |  |  |  |  |
| Diabetes, hypertension, IHD |  | ✓ |  | ✓ |
| Asthma |  | ✓ |  |  |
| Death |  | ✓ | ✓ | ✓ |
| Medical examination |  |  | ✓ |  |
| Stroke |  |  |  | ✓ |
| **Bloods (plasma)** |  |  |  |  |
| WBC, RBC, Hb, Haematocrit, platelet count, lymphocyte^2^, vitamin B12, folate, ferritin, OGTT | ✓ |  |  |  |
| CBC |  | ✓ | ✓ |  |
| **SES** |  |  |  |  |
| Education | ✓ | ✓ |  |  |
| Occupation |  |  | ✓ |  |
| BMI: body mass index; MUAC: mid-upper arm circumference; BP: blood pressure; IHD: Ischaemic heart disease; WBC: white blood cells; RBC: red blood cells; Hb: haemoglobin; MCV: mean corpuscular volume; MCH: mean corpuscular haemoglobin; MCHC: Mean corpuscular haemoglobin concentration; RDW: red cell distribution width; MPV: mean platelet volume; PDW: platelet distribution width; OGTT: oral glucose tolerance test ; CBC: complete blood count; SES: socioeconomic status  ^1^Circumference, ^2^Count and % | | | | |

**Table S3:** Laboratory investigations available for MAS 3

| **Test** | **Participant** | **Sample used** | **Timepoint** | | | | | | **Method(s) used** | **Location of analysis** |
| --- | --- | --- | --- | --- | --- | --- | --- | --- | --- | --- |
|  |  |  | **Baseline^¶^** | **Gestational week 18** | **Gestational week 28** | **Child age 6 years** | **Child age 12 years** | **Child age 18 years** |  |  |
| **Albumin** | Offspring | Plasma |  |  |  | ✓ | ✓ | ✓ | Spectrophotometry (bromocresolgreen) | 1 |
|  |  |  |  |  |  |  |  |  |  |  |
| **CBC** | Mother | Blood |  | ✓ | ✓ | ✓ | ✓ |  | Cell counter | 1 |
|  | Father | Blood |  | ✓ | ✓ | ✓ | ✓ |  | Cell counter | 1 |
|  | Offspring | Blood |  |  |  | ✓ | ✓ | ✓ | Cell counter | 1 |
|  |  |  |  |  |  |  |  |  |  |  |
| **Creatinine** | Offspring | Plasma |  |  |  | ✓ | ✓ | ✓ | Photometry | 1 |
|  |  |  |  |  |  |  |  |  |  |  |
| **Ferritin** | Mother | Serum |  | ✓ | ✓ |  |  |  | Photometry | 2 |
|  | Father | Plasma | ü |  |  |  |  |  | ELISA | 1 |
|  | Offspring | Plasma |  |  |  | ✓ | ✓ | ✓ | ELISA | 1 |
|  |  |  |  |  |  |  |  |  |  |  |
| **Folate** | Father | Plasma | ✓ |  |  |  |  |  | MBA | 1 |
|  | Offspring | Plasma |  |  |  | ✓ | ✓ | ✓ | MBA | 1 |
|  |  |  |  |  |  |  |  |  |  |  |
| **OGTT** | Mother | Plasma |  | ✓ | ✓ |  |  |  | GOD-POD | 1 |
|  | Father | Plasma | ✓ |  |  |  |  |  | GOD-POD | 1 |
|  | Offspring | Plasma |  |  |  | ✓ | ✓ | ✓ | GOD-POD | 1 |
|  |  |  |  |  |  |  |  |  |  |  |
| **H. pylori** | Offspring | Plasma |  |  |  | ✓ | ✓ | ✓ | ELISA | 1 |
|  |  |  |  |  |  |  |  |  |  |  |
| **hS-CRP** | Offspring | Plasma |  |  |  | ✓ | ✓ | ✓ | ELISA | 1 |
|  |  |  |  |  |  |  |  |  |  |  |
| **Red cell folate** | Mother | Blood |  | ✓ | ✓ |  |  |  | MBA | 2 |
|  |  |  |  |  |  |  |  |  |  |  |
| **Total protein** | Offspring | Plasma |  |  |  | ✓ | ✓ | ✓ | Biuret | 1 |
|  |  |  |  |  |  |  |  |  |  |  |
| **Vitamin B12** | Mother | Blood |  | ✓ | ✓ |  |  |  | MBA | 3 |
|  | Father | Plasma | ✓ |  |  |  |  |  | MBA | 1 |
|  | Offspring | Plasma |  |  |  | ✓ | ✓ | ✓ | Microbiological assay (colistin sulfate-resistant strain of L. Leichmanii.) | 1 |
| ^¶^ Baseline visit for mothers was the final visit before two successive missed menstrual periods were reported. Baseline visit for fathers occurred during either of the mother’s pregnancy visits.  1) Diabetes unit, KEMHRC, India; 2) Hematology Laboratory, Southampton General Hospital (Southampton, U.K.); 3) Bergen, Norway  CBC: complete blood count; OGTT: oral glucose tolerance test ; H. pylori: Helicobacter pylori antibodies; hS-RP: highly sensitive C-reactive protein; ELISA: Enzyme-linked immunosorbent assay; MBA: Microbiological assay (chloramphenicol-resistant strain of L. Casei); L. Casei: Lacticaseibacillus Casei; GOD-POD: Glucose Oxidase Peroxidase; L. Leichmanii: Lactobacillus Leichmanii  An additional four blood investigations for children (complete blood count, folate, vitamin B12, glucose) are also available at age 17 years. | | | | | | | | | | |

**Table S4:** Baseline cohort characteristics (fathers and household)

| **Variables** | **Total sample (N)** | **Mean (SD) / n (%)** |
| --- | --- | --- |
| **Fathers (**baseline) |  | **Mean (SD) / n (%)** |
| Height (cm) | 675 | 164.69 (6.06) |
| Weight (kg) | 681 | 52.85 (7.89) |
| BMI (kg/m^2^)^1^ | 674 |  |
| Underweight |  | 266 (39%) |
| Normal |  | 377 (56%) |
| Overweight |  | 31 (4.6%) |
| Obese |  | 0 (0%) |
| **Household** (baseline) |  | **n (%)** |
| SES^4^ | 709 |  |
| Upper class |  | 172 (24%) |
| Upper middle class |  | 390 (55%) |
| Middle class |  | 12 (1.7%) |
| Lower middle class |  | 127 (18%) |
| Lower class |  | 8 (1.1%) |
| Occupation (head of household) | 707 |  |
| Labour |  | 29 (4.1%) |
| Non-formal employment |  | 63 (8.9%) |
| Traditional occupation (hereditary profession) | | 484 (68%) |
| Business |  | 63 (8.9%) |
| Owner cultivator |  | 55 (7.8%) |
| Service (Formal employment) |  | 13 (1.8%) |
| Education level (head of household) | 707 |  |
| No formal education (unable to read/write) |  | 170 (24%) |
| Can read and write |  | 46 (6.5%) |
| Primary schooling | | 318 (45%) |
| Secondary schooling | | 24 (3.4%) |
| Junior college |  | 126 (18%) |
| Graduate |  | 23 (3.3%) |
| Number of adults in household | 707 |  |
| <= 5 |  | 244 (35%) |
| > 5 |  | 463 (65%) |
| SD: standard deviation; BMI: body mass index; SES: socioeconomic status  ^1^Calculated using World Health Organisation BMI categories: underweight (BMI<18 kg/m^2^); normal (BMI ≥18.5 kg/m^2^); overweight (BMI ≥25 kg/m^2^); obese (BMI ≥ 30 kg/m^2^).  ^4^Household SES was assessed using a questionnaire developed for rural populations in India [35]. | | |

**MAS 3 Project- Acknowledgments and contributions**

The following details are prepared to present role(s) of researchers/team members who have contributed for the two grants as mentioned below: (a) MAS 3 research grant submitted to the Global Challenges Research Fund scheme of 2020-21 at the University of Surrey, Guildford, UK, (b) MAS 3 PhD studentship grant submitted to the Faculty Funded Studentship Scheme 2021-22 at the University of Surrey**,** Guildford, UK.

*MAS 3 research grant (2020-21)*

Title: Maharashtra Anaemia Study Phase 3 (MAS3): Longitudinal study of nutritional, economic, and environmental impacts on anaemia, physical and cognitive development in Indian children from birth to age 18 years, nested within ‘The Pune Maternal Nutrition Study’ (PMNS). The MAS 3 project was conceptualised by Dr Anand Ahankari, Dr Andrew Fogarty and Prof Chittaranjan Yajnik, who worked together since the early stages to develop, design, and enhance project model. Further team members were contacted to request to join the team to offer their expertise to advance project design. These included Prof Laila Tata, Prof Charles Marshall, Prof Sumantra Ray, and Dr Sarah Bath. Prof Tata has expertise in large database analysis along with previous project supervision and experience in anaemia (conceptualisation, design and publication of MAS 1 and MAS 2 with Ahankari and Fogarty), adolescent health, maternal health and will provide advice on these areas to advance work through MAS 3 initiative (research as well as studentship grant).

Prof Marshall has expertise in cognitive development related research and provided inputs to develop these aspects into the grant and will also provide advice on this area for further work such as data analysis, interpretation, and dissemination for cognitive development/outcome related work. Prof Ray and Dr Bath have expertise in nutrition research and provided inputs on these aspects. Prof Yajnik evaluated the overall project from data availability aspects following discussions and inputs from Dr Ahankari from project management, finance, and implementation perspectives. Dr Fogarty has reviewed the entire project from clinical epidemiology and global health perspectives to support further development. All researchers named above reviewed the project grant submission and provided inputs considering their expertise. The project implementation work was completed in 2021, where Dr Ahankari worked with Prof Yajnik and MAS 3 project team based at the Diabetes Unit of the KEMHRC, Pune, India to complete activities within allocated funding and timeframe. Other than Dr Ahankari (primary investigators) all researchers named above were co-investigators on the MAS 3 grant.

*MAS 3 studentship grant (2021-22) and PhD programme (2022-26)*

*Title: Nutritional, economic, and environmental impacts on anaemia in Indian children from birth to age 18 years*

Dr Ahankari received permission from the researchers named above to develop a PhD studentship grant from the MAS 3 research grant, where Prof Nophar Geifman and Dr Sarah Bath were involved in project writing and submission. All researchers based outside the University of Surrey were external collaborators and provided a letter of support for the studentship project. Dr Kourosh Ahmadi from the University of Surrey was also involved as an internal collaborator considering his work on nutrition, Vitamin B12 and existing affiliation with Prof Yajnik. The MAS 3 PhD studentship is supervised by Dr Ahankari, Prof Geifman and Dr Bath. The PhD student (Ms Melissa Benavente) is a full-time postgraduate research student based in the School of Health Sciences at the University of Surrey to complete PhD studies between October 2022 and September 2026. All external collaborators will contribute to the analysis and interpretation of research output to advance Melissa’s PhD, and contribute to the publishing of the manuscripts that are subsequently generated.

*Research analysis and outputs*

PhD supervisors (Dr Ahankari, Prof Geifman, Dr Bath) will work on weekly/monthly basis with Melissa to support her studies. Once initial data analysis is completed for each proposed manuscript by Melissa and supervisors named above, initial results will be shared with Prof Yajnik, Dr Fogarty, and Prof Tata for their advice to improve the analysis further. Prof Marshall will be involved in cognitive development and related analysis work. Prof Ray and Dr Ahmadi will be involved in nutrition related research aspects of this work, which will be also supported by Dr Bath (one of the PhD supervisors). Melissa will primarily work on research objectives mentioned in her PhD project to lead on data analysis, interpretation, and dissemination activities supported by three supervisors, and all external collaborators will provide their specialised inputs and advice as mentioned above. All research manuscripts developed by Melissa for her PhD work will be shared with all individuals named in both grants mentioned above to offer them opportunities to be one of the authors on peer reviewed papers. Individual researcher will have a choice to decide based on their role and willingness and also availability at the time of conducting and publishing research work. Those who opt out later in the future, an email confirmation of the same would be required and their contribution will be mentioned in acknowledgment section(s) in published research papers. Authors contributions in manuscript will include information mentioned in this document in condensed version in line with journal guidelines. Dr Ahankari will support Melissa for outlined coordination and will work closely with all internal and external stakeholders. Additional research hypotheses will be developed by Dr Ahankari jointly with Dr Fogarty to advance work through MAS 3 dataset. This will involve Prof Yajnik and relevant researchers from the project depending on research hypotheses, scope, and availability of researchers to support such work. The decision will be made by Dr Ahankari following guidance from Dr Fogarty in line with good research practice guidelines.

Key universities and institutes where the research was conducted, implemented, and supported by will be acknowledged in all research outputs. Individual authors affiliation will be included in line with journal guidelines.

**PMNS Project- Acknowledgments and contributions**

The MAS 3 database is developed from the PMNS project initiated and implemented by Prof Yajnik. Each research paper published from the MAS 3 database will acknowledge contributions made by researchers and staff at the Diabetes Unit since its inception along with institutional support provided by university and organisations. A generic statement will be developed in agreement with Prof Yajnik to include in all research papers as standard to thank PMNS project team, involved staff, funders, and organisations
