# Supplementary material for: Cohort profile: the Maharashtra Anaemia Study 3 (MAS 3)—a maternal-child cohort study up to age 18 years in India
Source: BMJ Open. 2025 Oct 28;15(10):e104184. doi: 10.1136/bmjopen-2025-104184 (PMC12570902; doi:10.1136/bmjopen-2025-104184)
Supplement: Supplementary file 2 [file bmjopen-2025-104184.reviewer_comments.pdf]

PEER REVIEW HISTORY

BMJ Open publishes all reviews undertaken for accepted manuscripts. Reviewers are asked to complete a checklist review form and are provided with free text boxes to elaborate on their assessment. These free text comments are reproduced below.

ARTICLE DETAILS

Title (Provisional)

Cohort profile of the Maharashtra Anaemia Study 3 (MAS 3): a maternal-child cohort study up to age 18 years in India

Authors

Benavente, Melissa Toni; Geifman, Nophar; Bath, Sarah C.; Ahmadi, Kourosh R; Fogarty, Andrew W; Marshall, Charles; Ray, Sumantra; Tata, Laila J.; Yajnik, Chittaranjan; Ahankari, Anand

---

VERSION 1 - REVIEW

**Reviewer**	**1**
**Name**	**Cardoso, Marly**
**Affiliation**	**Universidade de São Paulo, Nutrition**
**Date**	**30-May-2025**
**COI**	**None**

This manuscript describes the overall profile of the Maharashtra Anaemia Study 3 (MAS3) cohort study. While the study design is appropriate and relevant to the topic, the manuscript requires major improvements in its current form. Below are some recommendations:

1. ABSTRACT:

Purpose: First, clearly state the aims of the MAS3 cohort, followed by its design as a nested cohort study within the PMNS.

Findings to date: In the Participants section, clarify the baseline data of MAS3. Additionally, attrition rates should be reported for each follow-up timepoint.

2. STRENGTHS AND LIMITATIONS:

The recruitment process involving 6 villages is not mentioned in the Abstract—please add this detail either in the Abstract or this section. The statement, "meaning findings are unlikely to be generalisable to urban populations," should be reworded for clarity. Does this imply that the study was conducted exclusively in rural areas?

3. INTRODUCTION:

The literature review lacks discussion on distal determinants (socioeconomic factors) of anemia during pregnancy and childhood.

Figure 1 appears in this section but should instead be included in the MAS3 cohort description, with additional details: i) attrition rates at each follow-up survey relative to eligible baseline participants; ii) a brief explanation of the number of non-participants and reasons for attrition.

4. MAS3 COHORT DESCRIPTION:

Table 1 should specify in its title that it presents baseline cohort characteristics.

Tables 2 and 3 contain redundant data on offspring—consider consolidating them into a single table.

Figure S1 should be moved to the main text rather than remaining a supplementary figure.

5. SES (Socioeconomic Status):

Were data collected on maternal education, occupation, and ethnicity? These factors are well-documented determinants of anemia in pregnancy and childhood and should be included if available.

**Reviewer**	**2**
**Name**	**Edison, E**
**Affiliation**	**Christian Medical College, Haematology**
**Date**	**21-Jul-2025**
**COI**	**None**

The authors present an important study that gives a chronic profile of anemia and micronutrient deficiencies in both mothers and their offspring, with expected follow-up data extending to 18 years.

Without the results it is just a description of the study and the study cohort. There are no real findings here.

1. Figure 1: While 710 mothers are reported at both gestational week 18 and delivery, the number drops to 700 at week 28. Could the authors explain this drop? Was it due to missed visits, early delivery, or other reasons?

2. Figure 1: There appears to be an inconsistency between the number of mothers reported at each follow-up time point and the categorisation provided under "data availability across the long-term follow-up visits. Please clarify.

VERSION 1 - AUTHOR RESPONSE

Reviewer 1 comments		Authors' response
1.	Abstract<br>Purpose: First, clearly state the aims of the MAS3 cohort, followed by its design as a nested cohort study within the PMNS.<br>Findings to date: In the Participants section, clarify the baseline data of MAS3. Additionally, attrition rates should be reported for each follow-up timepoint.	As advised, the following changes have been made to the abstract.<br><br>Purpose: Cohort aims have been stated before the nested structure of the cohort.<br><br>Findings to date: As advised, clarification has been provided regarding the baseline data for women recruited and children born into the study. The attrition rates for children at each of the long-term follow-up visits has been added to the abstract.<br><br>*"At the 6-, 12-, and 18-year follow-up visit, data were available for 706 (99%), 689 (97%), and 694 (98%) children."*
2.	Strength and limitations<br>The recruitment process involving 6 villages is not mentioned in the Abstract—please add this detail either in the Abstract or this section. The statement, "meaning findings are unlikely to be generalisable to urban populations," should be reworded for clarity. Does this imply that the study was conducted exclusively in rural areas?	As advised, the recruitment of participants from six villages has been added to the abstract and clarification regarding the rural setting of the study has been added to the strengths and limitation section which follows the abstract.<br><br>*"Recruitment occurred in six villages, approximately 50 km from Pune city in India, in a predominantly rural setting."*
3.	Introduction<br>The literature review lacks discussion on distal determinants (socioeconomic factors) of anemia during pregnancy and childhood.<br><br>Figure 1 appears in this section but should instead be included in the MAS3 cohort description, with additional details: i) attrition rates at each follow-up survey relative to eligible baseline participants; ii) a brief explanation of the number of non-participants and reasons for attrition.	As advised, the introduction has been amended to include risk factors of anemia.<br><br>*"Risk factors for childhood anemia include low socioeconomic status, often indicated by household wealth or mother's educational attainment or occupation, vegetarian diet, onset of menarche (girls), and maternal anemia [2,4,5]."*<br><br>As advised, Figure 1 has been moved to the section MAS 3 Cohort Description. In addition, attrition rates have been added along with information on attrition such as whether a participant missed a visit, was lost to follow-up, or had died. Please see page 2 of Figures file.
4.	MAS 3 cohort description<br>Table 1 should specify in its title that it presents baseline cohort characteristics.<br>Tables 2 and 3 contain redundant data on offspring—consider consolidating them into a single table.<br>Figure S1 should be moved to the main text rather than remaining a supplementary figure.	As advised, the following changes have been made.<br><br>- Table 1 title has been changed to "Baseline cohort characteristics".<br>- Please note that Table 2 is reporting maternal data collection whilst Table 3 is reporting child data collected. The authors have made the decision to move Table 2 (Maternal data collected at the pre-pregnancy (baseline) visit and during two points in pregnancy for MAS3) to supplementary material (now Table S2) to avoid confusion to the reader.<br>- Figure S1 has been moved from the supplementary material to the main text and renamed to Figure 2.

5.	<u>SES (Socioeconomic Status):</u><br>Were data collected on maternal education, occupation, and ethnicity? These factors are well-documented determinants of anemia in pregnancy and childhood and should be included if available.	We confirm that mother's educational attainment in years was collected, along with occupation. As advised, details on education and occupation have been added to socioeconomic status section: please see below. Mother's ethnicity was not collected, however, caste of the family was collected as part of the standardised questionnaire used to determine overall household socioeconomic status and has already been listed in this section.<br><br>*"Data relating to parental education and occupation were also collected. At the parental baseline (pre-pregnancy) visit and 6-year follow-up, education in years was reported for both mothers and fathers. At the 12- and 18-year follow-up visits, the occupation of mothers and fathers was collected."*
**Reviewer 2 comments**		**Authors' response**
6.	Figure 1: While 710 mothers are reported at both gestational week 18 and delivery, the number drops to 700 at week 28. Could the authors explain this drop? Was it due to missed visits, early delivery, or other reasons?	As advised, the participant flowchart has been expanded to include additional information on attrition. Please see page 2 of Figures file.
7.	Figure 1: There appears to be an inconsistency between the number of mothers reported at each follow-up time point and the categorisation provided under "data availability across the long-term follow-up visits. Please clarify.	Thank you for your query. The authors have opted to remove this section from the footnote following the addition of information relating to participant attrition which, we believe, sufficiently highlights that the data are unbalanced i.e. participants varied in the number of visits they attended.

VERSION 2 - REVIEW

**Reviewer**	**1**
**Name**	**Cardoso, Marly**
**Affiliation**	**Universidade de São Paulo, Nutrition**
**Date**	**23-Sep-2025**
**COI**	

This manuscript describes the overall profile of the Maharashtra Anaemia Study 3 (MAS3) cohort study. While the study design is appropriate and relevant to the topic, the manuscript requires major improvements in its current form. Below are some recommendations:

1. Abstract:

- clarify the MAS3 study design, including information on “This paper introduces the MAS 3 cohort which consists of data collected from the participants in the PMNS study from the antenatal period to offspring at 18 years of age (1996-2014)”.

- participants and findings to date: provide information on MAS3 results; the current version mentioned data from the PMNS main cohort.

2. STRENGTHS AND LIMITATIONS OF THIS STUDY in the first page

- The authors should include limitations of the MAS3 cohort in this section.

3. Introduction:

There is no mention to overall determinants and effectiveness of public health interventions to prevent anemia in childhood (mainly in the pregnant women and young children).

4. Figure 1: please add information on i) attrition rates at each follow-up survey relative to eligible baseline participants; ii) a brief explanation of the number of non-participants and reasons for attrition.

5. Findings to date: The authors have mentioned “Findings from the MAS 3 Study are yet to be published, though findings from PMNS have been widely published”. Although, I think that for this manuscript, findings from the MAS3 should be at least summarize for a cohort profile article.

VERSION 2 - AUTHOR RESPONSE

**Reviewer 1 comments**		**Authors’ response**
8.	<u>Abstract</u><br><br>- Clarify the MAS3 study design, including information on “This paper introduces the MAS 3 cohort which consists of data collected from the participants in the PMNS study from the antenatal period to offspring at 18 years of age (1996-2014)”.<br><br>- Participants and findings to date: provide information on MAS3 results; the current version mentioned data from the PMNS main cohort.	As advised, we have amended the abstract to add further clarity to the study design.<br><br>*“This paper introduces the MAS 3 cohort which consists of data collected from the participants in the prospective Pune Maternal Nutrition Study from the antenatal period to children at 18 years of age (1996-2014) in the Maharashtra state, India.”*<br><br>We have clarified in the sub-heading “Participants” that the final number of participants included in the MAS cohort is 710. Likewise, the cohort characteristics reported under the sub-heading “Findings to date” relate to the final cohort of participants included in the MAS 3 cohort (n = 710). To make this clear to the reader, we have stated this.<br><br>*Participants*<br>*“Recruitment of 2466 married non-pregnant women, and their husbands, took place between June 1994 and April 1996 in six villages, approximately 50 km from*

		Pune city in India. Women were followed up monthly to identify those who became pregnant. A total of 797 pregnant women were followed up for data collection at or near gestational week 18 and 28, with further data collection for women and children occurring within 72 hours of delivery, for both live and stillbirths. Of the 797 women, 710 were included in the MAS 3 cohort and long-term follow up of children occurred at 6-, 12-, and 18-years of age."<br><br>*Findings to date*<br>*"In the MAS 3 cohort, most mothers (73%) were aged between 18-25 years at the time of their final pre-pregnancy visit (baseline) and half (55%) belonged to families of middle-upper socio-economic status (SES). At the children's baseline (birth) visit, children had a mean birthweight of 2,630 g (SD: 376), with one third (31%) of low birthweight (LBW). At the 6-, 12-, and 18-year follow-up visit, data were available for 706 (99%), 689 (97%), and 694 (98%) children."*
9.	<u>Strengths and limitations of this study</u><br><br>- The authors should include limitations of the MAS3 cohort in this section.	Thank you for raising this query. As advised, two limitations have been included in this section.<br><br>*"Recruitment occurred in six villages, approximately 50 km from Pune city in India, in a predominantly rural setting which is unlikely to be representative of Maharashtra state or India as a whole.*<br><br>*Due to the lack of formal education within the study population and high rates of illiteracy, all questionnaires were completed by field workers, potentially increasing the risk of interviewer bias which should be considered."*
10.	<u>Introduction</u><br><br>There is no mention to overall determinants and effectiveness of public health interventions to prevent anemia in childhood (mainly in the pregnant women and young children).	As advised, we have cited a number of national cross-sectional surveys which are conducted in India and used to assess the effectiveness of public health interventions.<br><br>*"The NFHS, along with other nationally representative surveys conducted in India, such as the Comprehensive National Nutrition Survey (CNSS) [6] and District Level Household Survey (DLHS) [7], are widely used to provide regular anaemia prevalence estimates at the national and state level. In doing so, these surveys provide policy makers with evidence as to the effectiveness of state and national anaemia prevention strategies."*
11.	<u>Figure 1</u><br>Please add information on i) attrition rates at each follow-up survey relative to eligible baseline participants; ii) a brief explanation of the number of non-participants and reasons for attrition.	Attrition rates have been added to figure 1 to display the percentage of participants who did not attend a given visit. As now detailed, reasons for missed visits and those lost to follow-up are unknown, a footnote has been added in the figure legend to state this.

12.	Findings to date<br><br>The authors have mentioned "Findings from the MAS 3 Study are yet to be published, though findings from PMNS have been widely published". Although, I think that for this manuscript, findings from the MAS3 should be at least summarize for a cohort profile article.	Thank you for your comment. As we had included a sub-heading titled "MAS 3 cohort description", as well as the sub-heading "MAS 3 cohort characteristics", we have opted to move the summary of the cohort characteristics under "Findings to date" to avoid duplication and to address your comment. Please see page 15-16.
